# The relationship between peak inspiratory flow and hand grip strength measurement in men with mild chronic obstructive pulmonary disease

**DOI:** 10.1186/s12890-022-01858-7

**Published:** 2022-02-17

**Authors:** Takahiro Tsuburai, Yuko Komase, Hajime Tsuruoka, Baku Oyama, Hiromi Muraoka, Naoya Hida, Takayuki Kobayashi, Shinya Matsushima

**Affiliations:** 1 Department of Respiratory Medicine, St. Marianna University School of Medicine, Yokohama City Seibu Hospital, Yasashi-chou 1197-1, Asahi, Yokohama, Kanagawa 241-0811 Japan; 2 Department of Rehabilitation, St. Marianna University School of Medicine, Yokohama Seibu Hospital, Yokohama, Japan

**Keywords:** COPD, Hand grip strength, 6-min walk test, Peak inspiratory flow

## Abstract

**Background:**

Chronic obstructive pulmonary disease (COPD) decreases quality of life and muscular strength. Inspiratory flow is important for inhalants in the bronchi but is complicated to measure in routine practice. We hypothesized that hand grip strength (HGS) would correlate with inhalation rate in patients with mild COPD.

**Methods:**

The COPD patients were recruited at the St. Marianna University School of Medicine, Yokohama Seibu Hospital, from 2015 to 2018. We measured peak inspiratory flow (PIF) through an In-Check flow meter attached with Diskus [PIF(D)] and Turbuhaler [PIF(T)] inhalers. The 6-min walking test (6MWT), and the fraction of exhaled nitric oxide (FENO), spirometry, HGS, or forced oscillation technique (FOT) parameters were measured.

**Results:**

Forty-four subjects were enrolled. All were men, with a mean age (± SD) of 77.8 ± 9.36 years. Thirty-nine patients had mild COPD. PIF(D) was 110 (80, 140) L/min (median, interquartile range), PIF(T) was 80 (70, 90) L/min, and HGS was 28.7 (13.8, 43.6) kgf. PIF(D) and PIF(T) were significantly correlated (r = 0.443, *p* = 0.003). PIF(D) was significantly correlated with age (r = − 0.327, *p* = 0.030) and HGS (r = 0.326, *p* = 0.031). PIF(T) was significantly correlated with age (r = − 0.328, *p* = 0.030), FVC (r = 0.351, *p* = 0.019), 6MWT distance (r = 0.392, *p* = 0.011), and HGS (r = 0.328, *p* = 0.030).

**Conclusion:**

HGS might be more useful for predicting PIF than other parameters. Also, elderly COPD patients need to be taught inhaled methods carefully.

## Introduction

Chronic obstructive pulmonary disease (COPD) is caused by cigarette smoke or other hazardous substances and results in airflow obstruction [1]. COPD has a high prevalence and is often managed by a general physician. Treatment of COPD primarily involves inhaled drugs, and peak inspiratory flow rate is important to select devises. And also, the 6-min walk test (6MWT) is used to assess response to treatment, activity level, and prognosis in COPD patients [2–5]. However, it is difficult to measure the inhalation ratio or to perform a 6MWT in clinical situation of primary care clinics in Japan. Easy-to-understand indicators are required.

Recent studies showed that hand grip strength (HGS) reflects activity levels, and low HGS is associated with mortality [6]. In COPD patients, HGS is associated with the antioxidant indicators serum club-cell protein 16 (CC16) and total sialic acid, which also predict forced expiratory volume in 1 s (FEV1)/forced vital capacity (FVC) [7]. HGS is also associated with the distance walked in the 6MWT, frequency of COPD exacerbation, and inspiratory flow rate [8–10], but evidences in this area are insufficient.

We explored whether HGS is associated with inhalation rate in mild COPD and could therefore be a simple method of determining inhalation rates in COPD patients managed by general physicians.

## Methods

### Subjects

We recruited COPD patients from outpatients who visited St. Marianna School of Medicine, Yokohama City Seibu Hospital, from 2015 through 2018. We measured the peak inspiratory air velocity with In-Check. It was a coincidence that all the subjects became man, and this probably reflected the high prevalence rate of COPD in men.

We diagnosed COPD according to the COPD Guidelines for Diagnosis and Treatment Fifth Edition in Japan (2018): 40 years or older; over 20 pack-years of smoking history; cough, sputum, or dyspnea; and FEV1/FVC < 70% by spirometry after inhalation of a bronchodilator [1]. The stages of COPD were classified as follows depending on the state of airflow obstruction: Stage I, %FEV1 ≥ 80%; II, 50% ≤ %FEV1 < 80%; III, 30% ≤ %FEV1 < 50%; IV, %FEV1 < 30%. Patients were excluded when they had an obvious thoracic deformity, had acute exacerbation of COPD within 6 months when the assessment was performed, required treatment for a lower airway infection, or were unable to complete the 6MWT in room air due to hypoxia or difficulty walking. Asthma-COPD overlap (ACO) was diagnosed based on the JRS Guidelines for ACO Management 2018 [11], when a patient met any two or more of the following criteria: (1) fluctuating or episodic respiratory symptoms, (2) asthma since before age 40, (3) FENO > 35 ppb, or (4) any two or more of (i) concomitant perennial allergic rhinitis, (ii) airway reversibility (change in FEV1 of > 200 mL and 20%), (iii) peripheral eosinophils in blood (> 5% or > 300/µL), or (iv) elevated IgE or positive specific IgE to a perennial inhaled antigen.

The study was performed in accordance with the principles of the Declaration of Helsinki and was approved by the Ethics Committee of St. Marianna University School of Medicine (University No. 2883) on April 21, 2015. Written informed consent was obtained from all participants.

### HGS

HGS was measured with a Jamar hydraulic dynamometer (Preston, IL) twice alternately for the left and right hands with the patient standing.

### 6MWT

The 6MWT was conducted in accordance with the American Thoracic Society standards [5]. We stopped 6MWT when subject’s SpO2 was lower than 80% or their modified Borg Scale scored upper 5.

### FENO, FOT, and spirometry

Physiological laboratory technicians performed each measure according to the operation manual. The tests were performed in the order FENO, FOT, and spirometry to avoid having one test influence the results of the subsequent tests.

Spirometry measurements were carried out by one of our hospital’s experienced physiological technicians using a CHESTAC-8800 (Chest M.I., Inc., Tokyo, Japan) following the JRS Guidelines for Respiratory Function Testing [12]. FVC, %FVC, FEV1, FV1/FVC%, %FEV1, and spirometric peak inspiratory flow rate (PIFRs) were measured by spirometry. Diffusing capacity of the lungs for carbon monoxide (DLCO) was measured by having patients inhale CO-containing gas and exhale it 10 s later, measuring the concentration of exhaled air, and calculating the %DLCO and %DLCO divided by alveolar volume (%DLCO/VA).

FENO was measured with a NIOX-VERO (Chest M.I., Inc.). In accordance with the instruction manual, after maximum inhalation, patients were asked to exhale at 50 mL/s. Only samples for which measurable values of NO were detected were used for the analysis.

FOT measurements were carried out with a MostGraph-01 (Chest M.I., Inc.). While patients were engaged in stable resting ventilation, respiratory resistance at 5 Hz (R5) and 20 Hz (R20), respiratory reactance at 5 Hz (X5), resonant frequency (Fres), and the area of low reactance were measured, and their average values, values during expiration and inspiration, and the difference between the values during expiration and inspiration were evaluated.

### Inhalation flow rate

We attached either the Diskus or Turbuhaler adaptor to the In-Check flow meter (Clement Clark International, UK). The flow was measured three times, and the highest value was adopted. And also, we calculated predicted peak inspiratory flow of Diskus according to the method of Seheult et al. [13]. The relationship was described by the following equation; predicted PIF(D) = 0.139 * PIFRs − 0.257 * Age + 47.696.

### Intake muscle strength

The patients were measured using a respiratory dynamometer (CHEST, VITAL POWER KH-101). Maximum inspiration from the residual capacity was maintained for 3 s. The measurement was performed three times, and the maximum value was adopted.

### Statistical analysis

Statistical analysis was conducted using JMP version 14 (SAS, Japan). All parameters were expressed as frequencies or medians and interquartile ranges. Correlations between parameters were indicated in terms of Spearman’s correlation coefficient (r), with *p* < 0.05 regarded as significant. The multivariate analysis was used to investigate the relationships among PIF (D) or PIF (T) and other parameters.

## Results

**Fig. 1:**
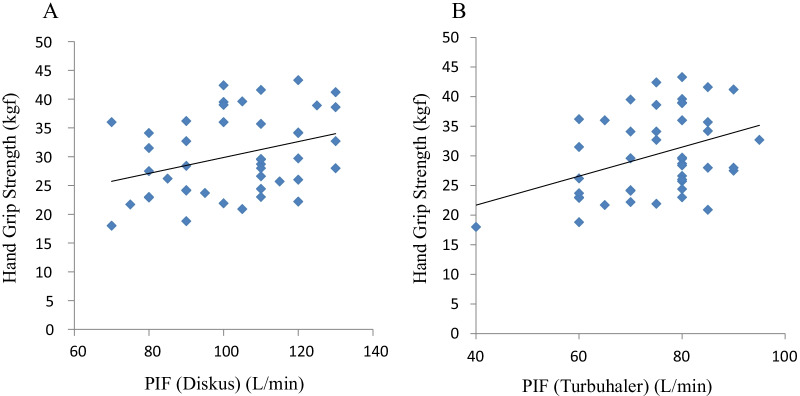
The retlationship between HGS and PIF (D) (**a**) or PIF(T) (**b**)

**Fig. 2:**
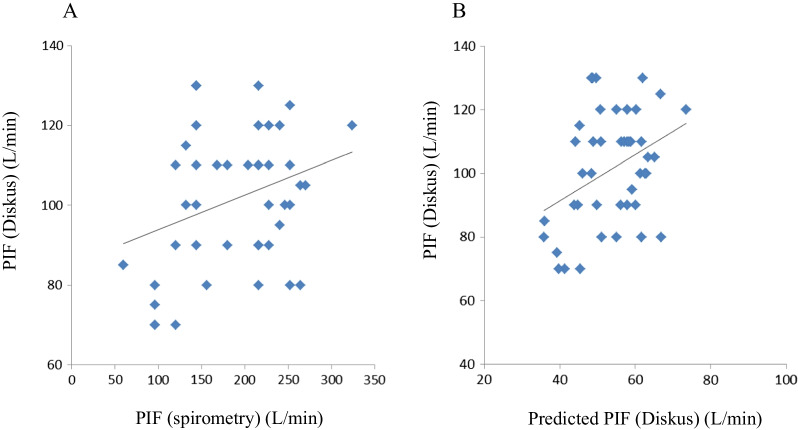
The relationship between PIF(D) and PIFRs (**a**) or predicted PIF(D) (**b**)

Forty-four patients were enrolled in the study, all of whom were men. Their backgrounds and parameters were showed at Table 1. The COPD stage of the patients in this study had comparatively mild (I: 28 patients (63.6%), II: 11 patients (25%), III: 5 patients (11.4%), and IV: none) and no patients had hypoxemia. The peak inspiratory flow (PIF) with the Diskus adaptor [(PIF(D)] was 110 (80, 140) L/min (median, interquartile range) and the PIF with the Turbuhaler [PIF(T)] was 80 (70, 90) L/min. PIF(D) of all subjects were above 60 (L/min), and PIF(T) of only one subject was below 60 (L/min). HGS was 28.7 (13.8, 43.6) (kgf). There was a significant correlation between PIF(D) and PIF(T) (r = 0.443, *p* = 0.003). Both PIF(D) and PIF(T) were correlated with age and HGS (Table 2, Fig. 1). PIF(T) was also significantly correlated with FVC (r = 0.351, *p* = 0.019) and the distance walked in the 6WMT (r = 0.392, *p* = 0.011) (Table 2). Moreover, there was significant relationship between age and HGS (r = − 0.667, *p* < 0.001). And also, the levels of PIF(D) were significantly correlated with PIFRs (Fig. 2a, r = 0.300, *p* = 0.047) and predicted PIF(D) by PIFRs (Fig. 2B, r = 0.367, *p* = 0.014).

**Table 1:** Subjects’ characteristics

No.	44
Sex (M/F)	44/0
Age	77.8 ± 9.36
BMI	23.3 ± 3.47
COPD stage (I/II/III/IV)	28/11/5/0
*Therapy*	
ICS + LABA + LAMA	7
ICS + LABA	20
LABA + LAMA	10
ICS + LAMA	3
ICS/LABA/LAMA	1/1/1
Diskus/Turbuhaler	12/5
FVC (L)	3.42 ± 0.79
%FVC (% of predicted)	110.5 ± 20.6
FEV_1_ (L)	1.88 ± 0.78
%FEV_1_ (% of predicted)	93.6 ± 33.6
FEV_1_/FVC (%)	54.6 ± 15.6
PIFRs (L/min)	187.3 ± 60.9
DLCO/VA (%)^†^	65.7 (30.7, 100.7)
Distance of 6MWT(m)^#^	442.0 ± 102.6
Hand grip strength (kgf)	28.7 (13.8, 43.6)
FENO (ppb)	17.0 (6.0, 34.0)
R5 (cmH_2_O/L/s)	3.31 (0.89, 5.73)
R20 (cmH_2_O/L/s)	2.32 (0.79, 4.11)
X5 (cmH_2_O/L/s)	− 0.53 (− 2.32, 1.26)
Fres (Hz)	7.73 (4.0, 16.84)
ALX (cmH_2_O/L/s*Hz)	1.75 (0, 11.9)
PIF(D) (L/min)	110 (80, 140)
PIF(T) (L/min)	80 (70, 90)
PImax (cmH_2_O)*	139.7 (94.4, 185)

The data are presented as means ± SD or median (interquartile range)

*6MWT* 6-min walking test, *PIF* peak inspiratory flow, *PImax* maximum inspiratory mouth pressure

N = 44 except *PImax (n = 35), ^#^6MWT (n = 41), and ^**†**^%DLCO/VA (n = 41)

**Table 2:** The relationship between PIF(D) or PIF(T) and other parameters

	PIF (D)		PIF (T)	
	r	*p*	r	*p*
Age	− **0.327**	***0.030***	**0.328**	***0.030***
BMI	0.030	*0.848*	0.088	*0.569*
FVC (L)	0.290	*0.056*	**0.351**	***0.019***
%FVC (% of predicted)	0.242	*0.114*	0.293	*0.054*
FEV_1_ (L)	0.282	*0.064*	0.191	*0.215*
%FEV_1_ (% of predicted)	0.101	*0.513*	0.076	*0.625*
FEV_1_/FVC (%)	0.125	*0.419*	− 0.029	*0.851*
PIFRs (L/min)	**0.301**	***0.047***	0.070	*0.640*
DLCO/VA (%)^†^	0.156	*0.329*	0.047	*0.769*
Distance of 6MWT (m)^#^	0.253	*0.111*	**0.392**	***0.011***
Hand grip strength (Kgf)	**0.326**	***0.031***	**0.328**	***0.030***
FENO(ppb)	0.004	*0.978*	0.484	*0.484*
R5 (cmH_2_O/L/s)	− 0.044	*0.775*	0.148	*0.338*
R20 (cmH_2_O/L/s)	− 0.046	*0.764*	0.135	*0.384*
X5 (cmH_2_O/L/s)	0.074	*0.631*	− 0.106	*0.435*
Fres (Hz)	− 0.053	*0.733*	0.126	*0.417*
ALX (cmH_2_O/L/s*Hz)	− 0.017	*0.911*	0.142	*0.357*
PImax (cmH_2_O)*	0.189	*0.278*	0.298	*0.082*

Factors in bold indicate *p* <0.05; Slash showed statistical data

r: Spearman rank correlation coefficient among parameters

N = 44 except *PImax (n = 35), ^#^6MWT (n = 41) or ^†^%DLCO/VA (n = 41)

But the levels of predicted PIF(D) were almost half of those of PIF(D).

Further to investigate the relationships between PIF(D) or PIF(T) and other parameters, we performed multiple regression analysis (Table 3). Age and HGS were not significantly associated with PIF(D)or PIF(T) independently.

**Table 3:** The multiple regression analysis among age, HGS and PIF(D) or PIF(T)

	PIF (D)		PIF (T)	
	B	*p*	B	*p*
Age	0.307	0.09	0.075	0.69
HGS	− 0.157	0.38	− 0.233	0.22

## Discussion

In male patients with mild COPD, higher PIF(D) and PIF(T) were associated with lower age and higher HGS. Although the relationship of PIF to age is a well-established theory, the correlation between inhalation flow rate and HGS in mild COPD patients is a new finding.

The level of inhalation flow is important for COPD management because it is associated with the distribution of inhaled dry-powder drugs. In a clinical situation, the type of inhalation drug is often selected with reference to the respiratory function and guidance on inhalation techniques. Seheult et al. were demonstrated that PIF(D) were correlated with PIFRs [13]. They also presented a prediction formula for PIF(D) based on PIFRs. In this study, the levels of PIF(D) were significantly correlated with PIFRs and predicted PIF(D) from PIFRs (Fig. 2), But PIFRs was not correlated with PIT(T), and the levels of predicted PIF(D) were almost half of those of measured PIF(D). It is a reasonable idea to create a PIF(D) prediction formula based on PIFRs, but the actual measured values may differ depending on the situation. It would be more convenient to have an objective, simple, and easy-to-understand index for predicting the inhalation flow rate.

Devices of dry powder inhaler are divided into low resistance and medium and high resistance, Diskus is low resistance inhaler, and Turbuhaler is a inhaler categorized in medium and high resistance [14]. If PIF exceeds 60 (L/min), any device will have sufficient flow rate for treatment. In this study, effective inhalation was performed in all cases. If HGS exceeds 20 (kgf), it is assumed that effective inhalation for DPI is possible. In addition, multiple regression analysis did not find an independent association between age and grip strength with PIF(D) or PIF(T). Although it is thought to reflect the decrease in activity due to aging, one recent study [10] showed that there was significant correlation between HGS and PIF independently of gender and age. It is considered that the number of subjects in this study is small. Frohnhafer et al. showed that HGS and inhalation flow velocity are directly correlated, and effective inhalation is not possible in patients with COPD if their HGS is less than 10 (kgf) [10]. In our study, there were no patients with HGS below 10 (kgf), but our results are generally in agreement with those of this previous study. Most of the COPD managed by primary care physicians is mild, so that the results of this study are likely to be useful in primary care settings.

The inspiratory muscles, such as the diaphragm or external intercostal muscles are important for inhalation, but it is difficult to measure inspiratory muscle strength clinically. We measured maximum inspiratory mouth pressure (PImax) in 35 patients, but there was no significant relationship between PIF(D) or PIF(T) and PImax (Table 2). PImax is not measured in all cases and may affect the results, but this suggests that not only muscle strength but the timing of inspiration are required for predicting PIF. Although HGS reflects the muscle strength of the upper limbs and does not directly indicate the muscle strength of the lower limbs or inspiration, it does reflect the overall strength of muscles used in daily life. Thus, it is not surprising that the evaluation of muscle strength required for COPD management can be performed with HGS. In fact, there was one recent report showing significant correlation between 6MWT and HGS in COPD [8]. Although HGS has not yet been used in general medical care for COPD, because of its simplicity—it can be measured simply by grasping the grip-strength meter—it may become a useful evaluation method in the future.

A limitation of this study is that it included only a small number of men at a single institution. All the cases that happened to participate in this study happened to be male. This is due to the fact that men have more COPD. Men tend to have higher muscle strength than women, so that this study shows a tendency in men. Women’s muscle strength tends to be weaker than men and require new research about women. In addition, there were relatively many cases of ACO, which may affect the results. PImax, 6MWT, DLCO had not been measured in all cases, and may affect results. Diskus and Turbuhaler were old Devices, so that the results with new devices, such as Ellipta or Breezhaler, were unknown. As a cross-sectional study, the results cannot be used to determine causal relationships, which would require a multicenter prospective study.

## Conclusions

In conclusion, both PIF (D) and PIF (T) correlated with HGS and age. HGS predicted inhalation flow better than lung function tests and may predict therapeutic efficacy and prognosis.

## Data Availability

All data generated or analysed during this study are included in this published article and its supplementary information files.

## Abbreviations

6MWT: The distance of 6 min walking test
BMI: Body-mass index
COPD: Chronic obstructive pulmonary disease
DLCO: Diffusing capacity of the lungs for carbon monoxide
FENO: The fraction of exhaled nitric oxide
FEV1: Forced expiratory volume in 1 s
FOT: The forced oscillation technique
Fres: Resonant frequency
HGS: Hand grip strength
PIF: Peak inspiratory flow
PImax: Maximum inspiratory mouth pressure
